# Hormonal, clinical, and genetic profile of infertile patients with azoospermia in Morocco

**DOI:** 10.11604/pamj.2023.45.119.38249

**Published:** 2023-07-10

**Authors:** Chaymae Rochdi, Ibtissam Bellajdel, Anouar El Moudane, Soufiane El Assri, Samira Mamri, Hafsa Taheri, Hanane Saadi, Ali Barki, Ahmed Mimouni, Mohammed Choukri

**Affiliations:** 1 Maternal-Child and Mental Health Research Laboratory, Faculty of Medicine and Pharmacy, Mohammed First University, Oujda, Morocco,; 2 Medically Assisted Procreation Unit, Central Laboratory, Mohammed VI University Hospital Center, Oujda, Morocco,; 3 Obstetrics Gynecology Service, Mohammed VI University Hospital Center, Oujda, Morocco,; 4 Urology Service, Mohammed VI University Hospital Center, Oujda, Morocco

**Keywords:** Azoospermia, hormonal profile, chromosome, spermogram

## Abstract

**Introduction:**

azoospermia affects more than 10%-15% of infertile male subjects attending the infertility center. In Morocco, there have been no studies on male infertility with azoospermia. Thereby, our objective was to evaluate the clinical, hormonal, and genetic characteristics of infertile men with azoospermia in Morocco.

**Methods:**

we conducted a retrospective descriptive study performed with a convenience sample of 80 infertile men from 2021 to 2022, in the Assisted Reproductive Technology Unit of the Mohammed VI University Hospital Center in Oujda-Morocco. All patients with azoospermia were subjected to a quantitative hormone assay to evaluate the functionality of the sertolic and leydigial compartments. Human karyotyping and AZF microdeletion analysis are routinely performed in azoospermic patients.

**Results:**

the results show that the mean age of patients in the study was 45.7 ± 3.5 years. Primary infertility accounts for the majority, with a rate of 96% (n=77). There were 12 cases of azoospermia of secretory origin, 22 cases of excretory origin, and 3 of undetermined origin. Azoospermia was associated with hydrocele in 29% (n=27) of cases. The average levels of FSH, LH, testosterone, and inhibin B were 15.54 ± 5.5 mIU/mL, 7.71 ± 2.7 mIU/mL, 405.09 ± 6.13 ng/dl and 38.44 ± 5.13 pg/ml, respectively. The prevalence of chromosomal abnormalities was 30.7%. Of these, the sex chromosome aneuploidy with 47, XXY karyotype (Klinefelter syndrome) accounted for 11% (n=9). The incidence of microdeletions of azoospermia factors (AZF) was 9%, and AZFc deletion was the most common at the rate of 3%.

**Conclusion:**

our research shows that hydrocele, varicocele, and chromosomal abnormalities are the leading causes of azoospermia. In the Moroccan population, azoospermia is essentially of excretory origin.

## Introduction

Infertility affects between 10-18% of married couples in the reproductive age group globally [1], and it is estimated that between 40 and 50 percent of infertilities is caused by men [2]. Azoospermia is the most severe form of male infertility. Azoospermia is characterized by the total absence of spermatozoa in the ejaculate [3]. Centrifugation of a semen specimen at a speed of at least 3,000 g for 15 minutes at room temperature with a high-powered microscopic examination is required to confirm this diagnosis [4]. It affects less than 1% of men and about 10%-15% of patients consulting for infertility [5]. Azoospermia is a side effect of numerous incurable testicular disorders [6]. Azoospermia is the main indication for testicular biopsy. It is caused by two mechanisms: obstructive (OA, excretory) due to a hindrance in the reproductive system with normal spermatogenesis or non-obstructive (NOA, secretory) due to a defect in testicular sperm production [7]. The two types of azoospermia are distinguished based on a combination of clinical, spermiological, hormonal, ultrasound and genetic data. About 60% of azoospermic males will have NOA, making it the most prevalent form of the disease [8]. A small proportion of NOA patients may be helped by the development of intracytoplasmic sperm injection (ICSI), testicular sperm extraction, and microdissection to produce biological children [9]. In 1995, the first cases of pregnancies obtained by ICSI with sperm of testicular origin were reported, first in patients with obstructive azoospermia, then in patients with secretory azoospermia, and even in patients with Klinefelter syndrome [10]. The epidemiology of azoospermia is significant and merits investigation. Early diagnosis and screening programs will surely expedite timely and appropriate management. The objective of the present study was to evaluate the clinical, biochemical, and genetic results concerning 80 azoospermic men investigated in the Assisted Reproductive Technology Unit of Mohammed VI University Hospital Center in Oujda, Morocco. The identification of such factors will then provide the distinction between obstructive and non-obstructive azoospermia.

## Methods

**Study design and setting:** this is an observational retrospective study with epidemiological and diagnostic purposes of azoospermic patients at the center of medically assisted procreation over a year, from September 2021 to September 2022.

**Inclusion criteria:** the patient performed 2 semen samples but there was no sperm in the semen (azoospermia). Patients were fully evaluated for hormonal, clinical, and genetic characteristics.

**Exclusion criteria:** subjects without azoospermia after spermiological examination. Subjects who received radiotherapy, chemotherapy, or hormonal treatment were excluded.

**Participants:** this retrospective research includes 80 patients. All of them consulted for primary infertility due to azoospermia.

**Variables:** an individual survey form was developed to collect and save the information, and the data was entered and analyzed. The questioning made it possible to specify the socio-demographic characteristics of the patients, their lifestyle (exposure to heat, radiation), their dietary habits (smoking, etc.), and their medical and surgical history.

**Data sources:** all the examinations described are routinely done in our center for the evaluation of azoospermic patients.

**Semen analysis:** sperm analysis is the first examination performed in the case of male infertility. It is based on a semen sample collected by masturbation in the laboratory in a sterile container after a period of sexual abstinence of 2 days and then kept in the oven for 30 to 60 minutes for liquefaction before doing a semen analysis, which includes volume and potential of hydrogen (pH) values. Direct examination of the semen was done by placing a drop of undiluted semen in a Leja cell. Azoospermia was verified in at least two ejaculates, by pellet analysis after semen centrifugation (3000 g for 20 min). The 6^th^edition of World Health Organization criteria (WHO) should be followed when performing the semen analysis [11], and at least two semen samples collected more than two weeks apart should be evaluated.

**Hormonal evaluation:** all azoospermic patients benefit from hormonal assays to assess the functionality of the Sertoli and leydigial compartments, whose complex interactions are necessary for proper spermatogenesis. A hormonal profile using quantitative analysis of luteinizing hormone (LH), testosterone, follicle-stimulating hormone (FSH), prolactin (PRL), and inhibin B was carried out in all patients. The blood determination of FSH, LH, testosterone, and PRL was performed on 10 ml of peripheral blood collected in a dry tube and centrifuged within one hour by an Eppendorf 5810R centrifuge to obtain the plasma used for the determination of hormones in the biochemistry department by using Architect ci8200.

**Genetic screening:** a genetic workup was performed including a constitutional karyotype in standard resolution, and a PCR search for Y chromosome microdeletions of Sequence-Tagged Sites (STS) markers specific to the microdeletions of Azoospermia Factors (AZF) locus on the Y chromosome. Chromosomal analysis was performed on cultures of peripheral blood lymphocytes from our patients. For each patient, at least 30 well-spread metaphases were analyzed. The number of metaphases that were studied was increased to 50 metaphases after cytogenetic abnormalities were discovered in the patient. Using the PUREGEN DNA Purification Kit for the extraction of the DNA (Gentra system, Minneapolis, USA). The study of the STS markers of the Y chromosome was performed by multiplex PCR, using 3 pairs of primers specific to the AZFa (SY84, SY86), AZFb (SY127, SY134), and AZFc (SY254, SY255) regions.

**Physical examination:** an azoospermic man must undergo an extensive physical examination, which should be performed on him when he is standing and lying down in a warm environment. All participants underwent physical examinations by urologists. Exam results were documented using a standard form. To check out subjects with urological or reproductive disorders, secondary sexual characteristics, the possible presence of a varicocele or hydrocele, the position of the testis in the scrotum, and the consistency of the testis and epididymis were all investigated. A “Test-size” orchidometer was used to measure the testicular volume.

**Post-ejaculatory urinalysis:** patients having ejaculate quantities less than 1 ml underwent post-ejaculatory urinalysis. Sperm in urine samples from azoospermic or aspermic patients suggests retrograde ejaculation.

**Scrotal ultrasonography examination:** an ultrasound of the scrotal contents is routinely performed in azoospermic patients. During this examination, the urologist measures with precision the testicular volume (threshold value fixed at 16 ml for hypotrophy) and can identify cysts, spermatoceles, varicoceles, and lesions that can be difficult to find with conventional methods.

**Statistical methods:** statistical analysis was performed using the software package SPSS 20 (IBM) (Statistical Package for the Social Sciences). Student´s t-test was used for the statistical evaluation of the results. Data collected was analyzed and categorical variables were summarized as frequency and percentage while continuous variables were summarized as means, median and standard deviation (SD). The mean values of each parameter for the various groups or subgroups of azoospermic men were compared. A p-value less than 0.05 was considered to be statistically significant.

**Ethical consideration:** appropriate written informed consent according to the principles of the Declaration of Helsinki was approved by the local Ethical Committee of the Faculty of Medicine and Pharmacy of Oujda, Morocco.

## Results

Of the 1025 spermiological examinations performed in men coming for semen analysis, 80 (17.56%) were azoospermic patients. The patients were between the ages of 21 and 65, with a mean age of 45.7 ± 3.5 years. Among the patients, 34 (43%) were between the ages of 41 and 50. Infertility was primary in 77 (96%) of our patients and there were around 14 years of infertility on average. Drivers were the most represented profession in 26 (33%). Twenty-one (21 (27%)) of the patients were smokers and 11 (14%) were alcoholics, and 29 (37%) never smoked. The majority of our patients 59 (74%) had no family history (Table 1). The mean values of the hormone assays FSH, LH, Testosterone, and Inhibin B were respectively 15.54 ± 5.5 mIU/mL, 7.71 ± 2.7 mIU/mL 405.09 ± 6.13 ng/dl and 38.44 ± 5.13 pg/ml. Karyotype was performed in 80 cases. The abnormalities were: Klinefelter syndrome in 9 (11%) of cases and reciprocal translocation between the long arm of chromosome 14 and the short arm of chromosome 21 in 3 (4%) of cases. Fifty-seven (57 (72%)) of the patients had no karyotype abnormalities. A molecular study of the AZF locus, performed in 20 of 54 patients, revealed the existence of microdeletions in 5 cases (9%). AZFa microdeletions were the most frequent 3 (3%). For the clinical profile (scrotal echography), we found a hydrocele in 27(33%) patients, followed by varicocele in 20 cases (17%). Concerning the medical history, we found inguinal hernia in 15 patients (25%) and mumps in 10 patients (17%). On imaging, testicular volume was abnormal with a mean of 3ml. The sperm volume was normal with an average of 2.8 ml of ejaculate with extremes ranging from 0.1 ml to 5.4 ml. The average pH was basic at 8 (Table 2).

**Table 1:** sociodemographic characteristics of the azoospermic patients recruited from the fertility center in Mohammed VI University Hospital Center, Eastern region of Morocco, from September 2021 to September 2022 (N=80)

Variable	Frequency (n=80)	Percentage (%)
**Age (years)**		
21-30	5	7
31-40	32	40
41-50	34	43
≥ 50	12	10
**Type of infertility**		
Primary	77	96
Secondary	3	4
**Duration of infertility (years)**		
2-5	13	17
6-9	38	47
≥ 10	29	37
**Profession**		
Professionals	12	15
Skilled workers	30	38
Unskilled workers	38	47
**Education level**		
Illiterate	64	80
Primary	12	14
Secondary	4	6
**Substance use (%)**		
Smoking/tobacco	22	27
Alcohol	11	14
Previous smoking	19	24
Never smoked	28	37

**Table 2:** biochemical, genetic, and clinical characteristics of the azoospermic patients performed in the Mohammed VI University Hospital Center of Oujda-Morocco

Paraclinical and clinical characteristics	N	Percentage (%)	Mean±SD	Median	Min-Max
**Biochemical parameters**					
FSH (mIU/mL)	80		15.54 ± 5.5; (NV: 15-100)	17.4	3.78-31.02
LH (mIU/mL)	80		7.71 ± 2.7; (NV: 1-9)	9.81	1.67-17.95
Total testosterone (ng/dl)	80		405.09 ± 6.13; (NV: 236.5-997.9)	541.52	5.97-1077.07
Inhibine B					
Indosable	48	80%			
Dosable (pg/ml)	12	20%	38.44 ± 5.13; (NV: 100-160)	73	15-131
**Cytogenetics (karyotype)**					
Klinefelter's syndrome					
47, XXY homogeneous	5	9%			
47, XXY mosaic	1	2%			
Reciprocal translocation between the long arm of chromosome 14 and the short arm of chromosome 21	2	4%			
Absence of chromosomal abnormalities	72	86%			
**Y-chromosome microdeletion**					
AZFa	3	5%			
AZFa+ AZFc	1	2%			
AZFc	1	2%			
None	75	91%			
**Scrotal echography**					
Total testicular volume(ml)	80		8 ± 0.6; (NV: 16)	7.4	0.8 -14
Varicocele	20	17%			
Cryptorchidism	2	4%			
Testicular bursa trauma	19	15%			
Hydrocele	27	29%			
**Medical history**					
Mumps	10	17%			
Erectile dysfunction	2	4%			
Urogenital infection	8	14%			
Retrograde ejaculation	3	5%			
Inguinal or inguino-scrotal hernia	15	25%			
Diabetes mellitus	8	14%			
Thyroid disorder	4	7%			
**Spermogram**					
Volume of ejaculation (ml)	80		2.8 ± 1.2 (NV: > 1.6ml)	2.75	0.1-5.4
pH	80		8±0.7	7.8	7-8.6
Viscosity					
Very viscous	36	60%			
Little vicious	6	10%			
Liquefied	18	30%			

NV: Normal Value according to WHO 2021; FSH: follicle-stimulating hormone; LH: luteinizing hormone; pH: potential of hydrogen

Complementary examinations determined the secretory origin of azoospermia in 12 cases (15%) and the excretory origin in 22 cases (27%) and the origin of azoospermia remained undetermined in 46 cases (57%), due to the lack of clinical and biological arguments. A hormonal assessment was performed in 80 patients and showed elevated serum FSH levels in 12 cases of secretory azoospermia. Twelve (12) patients with secretory azoospermia had an abnormal karyotype. Scrotal ultrasound and/or testicular biopsy confirmed the clinical diagnosis in all cases, both examinations revealed testicular atrophy that was not suitable for andrological examination in 12 cases of secretory azoospermia. In 4 cases of secretory azoospermia, the physical examination revealed the existence of testicular ectopy. Twenty-four (24 (30%)) patients (10 cases of secretory azoospermia and 12 cases of excretory azoospermia) had a clinical or ultrasound varicocele (Table 3).

**Table 3:** biochemical, genetic, and clinical parameters according to the type of azoospermia in patients recruited from the fertility center in Mohammed VI University Hospital Center, Eastern region of Morocco, from September 2021 to September 2022 (N=80)

	Azoospermia (n= 80)				Total (%)
Results of clinical and paraclinical examinations	Secretory (non-obstructive) (n= 12)	Excretory (obstructive) (n= 22)	Indeterminate (n= 46)	P-value	
High follicle-stimulating hormone (FSH)	12 (100%)	_	_	< 0.001*	12 (17%)
Abnormal karyotype	11 (92%)	_	_	< 0.001*	11 (15%)
Y-Chromosome Microdeletion	3 (25%)	_	_	< 0.001*	3/56
Clinical and/or sonographic testicular hypotrophy/atrophy	12 (100%)	4 (18.18%)	7 (15.21)	< 0.05	23 (28.75)
Testicular ectopia	4 (33.33%)	2 (9.09%)	1 (2.17%)	< 0.003	7 (8.75%)
Clinical or ultrasound varicocele	10 (83.3%)	12 (54.5%)	2 (4.34%)	< 0.05	24 (30%)
Abnormal testicular biopsy	12 (100%)	_	_	< 0.001*	12 (15%)
Abnormal epididymal biopsy	2 (16.6%)	_	_	< 0.001*	2 (2.5%)
Disturbed seminal biochemistry	4 (33.3%)	12 (54.54%)	_	< 0.01	16 (30.18%)

* Student’s t-test

## Discussion

According to the WHO, infertility affects 8-12% of couples of reproductive age, and approximately one in six couples face infertility [12]. Recent years have seen a marked increase in the contribution of the male factor to infertility in relationships as a result of comprehensive assessments of male reproductive function and advances in diagnostic techniques [13]. The majority of male factor infertilities are characterized by a variety of semen quality, ranging from normal spermogram to no spermatozoa in semen [14,15]. The absence of all spermatozoa in two different centrifuged semen samples is defined as azoospermia [16,17]. It impacts less than 1% of men in the large population or 5-15% of infertile men [3]. The exploration of the azoospermic men is today of capital interest considering the assisted reproductive technologies essentially ICSI, which allow from now on men considered definitively infertile, to become fathers with their gametes [18].

Azoospermia can be of two types: secretory (NOA) with impaired or no spermatogenesis, or excretory (OA) with normal spermatogenesis but obstructed or impaired seminal tract from embryogenesis [7]. Based on a combination of clinical, spermiological, hormonal, ultrasonography and genetic data, the two types of azoospermia are distinguished. An attempt 'Pat surgical sperm collection is then proposed to couples who accept ICSI' [19,20]. Amongst azoospermic males, 40% will have OA. In OA, spermatogenesis is often normal but could also result from the following conditions: congenital bilateral vas deferens absence; obstruction of the ejaculatory and epididymal ducts; atresia of the seminal vesicles; various genitourinary tract infections that cause obstruction; or pelvic and inguinal procedures that result in a complete blockage, such as a bilateral vasectomy. In more than 90% of cycles with OA, sperm can be extracted by testicular sperm aspiration (TESA), percutaneous sperm aspiration (PESA), microsurgical sperm aspiration (MESA), or testicular sperm extraction (TESE) [21,22].

Age is a key determinant of a couple's capacity to conceive. The average age of the patients in our study was 42.42 years, ranging from extremes of 25 to 65 years. This outcome was following the data in the literature [23]. In our series, azoospermia was associated with primary infertility which may plead for a genetic and hormonal origin. Additionally, some lifestyle choices like smoking, drinking alcohol, and using recreational drugs may impact reproduction. No study has reported the prevalence of azoospermia in the eastern region of Morocco. Male reproductive function is mostly initiated and maintained by hormones. According to earlier research, men's circulating levels of particular reproductive hormones are related to semen quality indicators [7]. Follicle-stimulating hormone regulates spermatogenesis and is usually increased in secretory infertility. LH regulates blood androgen levels and testosterone has a fundamental trophic and functional effect on the seminal pathway [24].

It has also been proposed that measuring inhibin B and FSH in serum could replace evaluating semen quality or fecundity in epidemiologic research. These two hormones are regarded to be markers of spermatogenesis and Sertoli cell function in particular [25]. The etiology of azoospermia can be clarified using data from a full endocrine profile. It is important to collect hormonal profiles that show the levels of follicle-stimulating hormone, luteinizing hormone, prolactin, and inhibin B. Plasma levels of FSH, testosterone, and secondarily prolactin, report the testicular secretory status [26]. When studying azoospermic men, plasma FSH is considered to be the most significant hormone marker and should always be measured. Primary testicular failure is compatible with high FSH results. The FSH assay should be combined with other serum markers, such as inhibin B, which has a high predictive value in the evaluation of spermatogenesis deficits. In our study, the analysis of clinical and paraclinical data allowed us to identify some causes of azoospermia. Testicular biopsies from infertile males qualitatively evaluated for the extent of germ cell destruction revealed an inverse correlation with plasma FSH levels [27]. If the testosterone level is low, a total testosterone check should be performed with supplementation by free or bioavailable testosterone, LH, and prolactin.

Indeed, faced with a disorder of spermatogenesis, if the testicular volume is normal with a normal hormonal balance, the examination must eliminate an obstructive cause, particularly congenital bilateral agenesis of the vas deferens in cystic fibrosis [28]. This indicates the importance of the karyotype in the assessment of male infertility. Cytogenetics should also be part of our biological arsenal for the characterization of infertility. The results of the spermogram may lead to the performance of a karyotype and the search for Y chromosome microdeletion. Karyotype abnormalities are a major cause of male infertility. They can be subdivided into sex chromosome aneuploidy, structural abnormalities, or copy number variation. Approximately 15% of azoospermic patients have a karyotype abnormality, while this rate is 0.6% in the general population. These abnormalities may be inherited or de novo. Klinefelter's syndrome is the most common anomaly and is a major contributor to non-obstructive azoospermia, affecting approximately 10% of patients with azoospermia. Klinefelter's syndrome is the most common anomaly, resulting in hypogonadism and sterility [29]. In 80% to 90% of cases, the testicle is small, with very few or no sperm in the ejaculate. In our series, this syndrome was present in 6 azoospermic patients (11%).

According to WHO, the absence of a karyotype abnormality should prompt the treating physician to request a search for a Y-chromosome microdeletion. Microdeletions of the AZF locus on the Y-chromosome are present in 15-20% of idiopathic azoospermia and about 5-7% in idiopathic oligozoospermia [28]. Subsequent analysis of this region, called AZF for the Azoospermia Factor, allowed it to be separated into the AFZa, AZFb, and AZFc subregions. Microdeletions of the AZFa region are the least frequent (1-5%) and are usually associated with germ cell aplasia (isolated Sertoli cell syndrome). The molecular analysis of the Y chromosome should make it possible to determine the etiology of male infertility in about 10% of cases, to inform the patient of the transmission of the anomaly from father to son, and thus to improve the management of patients in the context of assisted reproduction [10]. Genetic exploration and counseling are nowadays essential steps in the assessment of sterility in cases of azoospermia [6].

In our study, the inguinal hernia was recorded in 25% of patients. Two percent (2%) of patients with untreated bilateral cryptorchidism develop azoospermia. Inguinal hernia is incriminated in infertility after surgical cure, which may be the cause of excretory azoospermia by iatrogenic obstruction of the vas deferens that cross the inguinal canal [14]. Through investigation of clinical diagnostic characteristics, obstructive azoospermia can be separated from nonobstructive azoospermia in the vast majority of patients. Seminal biochemistry is an important adjunct to the spermogram in the investigation of male infertility. In the case of obstruction, biochemistry allows us to determine the level and extent of obstructive phenomena which is the object of our next study.

## Conclusion

In male infertile patients with azoospermia, a history of varicocele and inguinal hernia, along with genetic abnormalities considered the main causes leading to non-obstructive azoospermia. The incidence of chromosomal abnormalities and the incidence of AZF deletion was quite high, suggesting the important role of screening testing for genetic abnormalities in infertile patients with non-obstructive azoospermia. A reliable basis for the differential diagnosis of secretory obstructive azoospermia is a biochemical examination of seminal markers.

### What is known about this topic

- *Infertility is a disease of the reproductive system and male infertility is a sacred subject in Morocco;*
- *Azoospermia is the most severe form of male infertility and no epidemiological research on the profile of the azoospermic patient in the eastern region of Morocco has been done to date;*
- *Clinical, hormonal, and genetic results concerning 80 azoospermic patients*.

### What this study adds

- *The identification of hormonal, clinical, and genetic factors will provide the distinction between obstructive and non-obstructive azoospermia;*
- *It is the first study in the eastern area of Morocco that provides a global view of the profile of azoospermic patients in this region;*
- *The prevalence of chromosomal abnormalities was 30.7% and the incidence of microdeletions of azoospermia factors (AZF) was 9%*.
